# Surgery

**Published:** 1904-08-20

**Authors:** 


### Progress in Medicine and Surgery.

### SURGERY.

(*Continued from page* 345.)

Fractures.—Burghard[2] in an address on the modern treatment of fractures, emphasises the importance of directing attention to the treatment of injuries of the soft parts as being of more importance than the care of the actual fracture. Coaptation of the fragments is, of course, necessary, but attempts at preserving absolute rigidity are quite unnecessary, even if they are possible. He advocates the use of a general anæsthetic in the first treatment of fracture; it quiets spasm, allows full muscular relaxation, and by abolishing pain greatly facilitates the task of bringing the bones into proper apposition and maintaining them so until splints are applied. Massage in all cases should be commenced at once, gentle effleurage for the first few days over the seat of fracture is soothing, increases the local circulation, and tends to aid the absorption of exudate. There are two distinct lesions in every fracture beside the injury to the bone: on the one hand tearing of muscular and tendinous structures in the vicinity of the fracture, on the other hand extravasation of blood. The latter is most effectually treated by massage, the former by passive movements commenced before these structures have commenced to become adherent to the seat of fracture, to one another, or to their tendon sheaths. The great object to be aimed at is to have a useful and supple limb, which can be used as soon as the bone is firm, instead of a firmly united bone with a stiff and painful limb. Especially does this principle apply to dislocations; the weakness of the joint is not due to the torn and injured capsule, it is the muscles around the joint that play the most important part in maintaining the joint surfaces in apposition. The treatment then is not to fix the joint so that the torn capsule may unite, but to exercise and improve the nutrition of the muscles and prevent the formation of adhesions. He deals in particular with the treatment of several common fractures. Sayre's method of treating fractures of the clavicle, which is most commonly used, does not commend itself to him as the most satisfactory he advocates the old "handkerchief" method. Two handkerchiefs are each folded round a roll of cotton wool until they are about the thickness of a perineal strap, a pad is placed in the axilla of the injured side, the rolled handkerchiefs are then looped one around each shoulder passing over the point of the shoulder and under the axilla, they are then fastened behind the back, so as to draw the shoulders well back, and the arm of the affected side is placed in a sling. In the recumbent position the arm should be fixed to the side. In three or four days the patient is allowed to practise underhand movements without the sling. Illustrating the importance of early movements and avoidance of too rigid fixation in Colles' fracture, he cites two cases, one of which occurred in a working-man. The patient was not treated at the time of the accident, but had rubbed and worked the injured part himself, and, at the end of eight weeks, when seen by the writer for the first time, the deformity was obvious; but there was neither pain nor stiffness, every joint of the hand was working well. The other case had been treated by splints for six weeks; when seen after that period the apposition was good and there was but little deformity, but the hand was stiff, extensive adhesions were present in the wrist and all the tendon-sheaths, and none of the fingers could be bent. Splints, of course, must be used to prevent deformity, but should be daily removed for massage and movement. The displacement in most cases of Potts' fracture can be readily reduced by flexing the knee and thigh to relax the calf muscles. Occasionally some structure, as the tendon of the tibialis posticus gets between the bone and so prevents reduction; open operation is then necessary. He advises the use of an external lateral splint, as most of the deformity is lateral and good position cannot be so well retained by the back splint so generally used. Gwilym G. Davis[3] considers that surgeons do not devote the attention they should to the treatment of fractures. No doubt the interest in fractures has waned in the last 20 years before the great advances in abdominal and other branches of surgery. Fractures are left to the assistants, are treated conservatively in time-honoured fixation appliances, and the results if bad are accepted with resignation. If good apposition cannot be obtained, operative measures should be early resorted to. Excessive callus is a cause of non-union, and the amount of callus is in direct ratio to the want of apposition, if the bones are in perfect apposition but little callus results. He cites a case in which he wired a spiral fracture of the tibia; after seven weeks he cut down to remove the wires, the apposition was perfect, and there was no trace of external callus. Though it is possible to treat fractures even of the thigh with a certain degree of success by ambulatory methods, he does not consider the results to be good. Swelling and pain are certain to arise when a recently-fractured limb is placed for any length of time in the upright position. Openshaw[4] describes a modification of Hessing's splint whereby a patient with a fractured leg or thigh can walk about from the beginning. He relates two cases of fractured leg and one of fractured thigh in which he used this apparatus with success. He claims that the nutrition of the muscles is maintained and the bones unite well, and the joints do not become stiff. He does not state whether swelling of the limbs occurred. R. W. Murray[5] calls attention to the erroneous but almost universal practice of putting up fractures of the leg on a back-splint with a vertical foot-piece. The leg is thus fixed in an unnatural position of internal rotation; the natural position of the leg of a person lying upon the back is external rotation, with the foot not vertical but turned outwards at an angle of about 70°, in the position of slight varus, and the toes slightly pointed downwards. When, therefore, the limb is on the ordinary splint, the thigh tends to rotate out into the natural position of rest, but the foot and lower fragment being firmly fixed cannot rotate out, so that there is a rotatory displacement between the fragments. He has splints made with a foot-piece set inclined outwards at an angle of 70°, with the same inclination forwards and a slight varus twist; the whole limb is thus fixed in the ordinary position of rest. He advocates the use of an anæsthetic in setting the limb, and in most cases where proper apposition cannot be obtained, early operation and wiring of the fragments. Late operation after much callus and adhesion have formed are attended with more difficulties, delayed union, and less satisfactory results. S. Lloyd[5] has obtained good results in several cases of ununited fracture of the neck of the femur by open operation; an anterior incision is made and the parts approximated, then through a second incision over the trochanter a wire nail is driven in, passing up the neck into the head of the bone. Separation of the acetabular epiphysis of the femur in adolescents is an accident of much commoner occurrence than is generally supposed; it is a condition that has only become recognised since the introduction of skiagraphy and operative treatment of fractures; many errors in diagnosis and treatment have been made in such cases. H. Betham Robinson[7] contributes an interesting article upon this subject. After injury the patient may be able to walk about, which fact leads the practitioner as a rule to exclude fracture. He recounts the history of six cases which have come under his notice. The diagnostic points to be observed are: (1) An injury to the hip, usually of a trivial nature, occurring in an adolescent, after which the patient begins to limp, pain not being a prominent feature; (2) deformity, the great trochanter is prominent and raised above Nélaton's line, the limb is everted; (3) limitation of movement especially internal rotation and abduction; (4) absence of the rigidity and tenderness of joint disease. A noticeable feature in these cases is that, previous to the accident, which is generally a slight one, the joint did not appear to be quite right, there were vague pains and limping for some time before. The author thinks this points to some pathological condition at work in the epiphysis, which predisposes to separation, perhaps of the same nature as that which gives rise to coxa vara in adolescents. In all the cases quoted the patient was able to get about after the injury. An important bearing of this condition is the liability of its being mistaken for early tuberculous hip disease. No doubt there may be difficulty if the case be seen for the first time at a considerable interval from the injury, but in early tuberculous disease no shortening would be found, and by the time this appeared other more certain evidences of joint disease would be present. As to treatment, if seen early he advises forcible abduction and inversion to attempt to bring the surfaces into apposition, and then to fix the limb in this position with plaster-of-Paris breeches. Later on, excision of the head of the bone is all that can be done.

[2] Lancet, Dec. 19, 1903. [3] Annals of Surg., May, 1904. [4] Lancet, March 12, 1904. [5] Brit. Med. Jour., Oct. 10, 1903. [6] Med. Record, Nov. 28, 1903. [7] Brit. Med. Jour., Oct. 10, 1903.

